# Supplementary material for: Lung clearance index in healthy volunteers, measured using a novel portable system with a closed circuit wash-in
Source: PLoS One. 2020 Feb 25;15(2):e0229300. doi: 10.1371/journal.pone.0229300 (PMC7041809; doi:10.1371/journal.pone.0229300)
Supplement: S1 Data — (DOCX) [file pone.0229300.s001.docx]

**Lung clearance index in healthy volunteers, measured using a novel**

**portable system with a closed circuit wash-in**

Alex Horsley, Amnah Alrumuh, Brooke Bianco, Katie Bayfield, Joanne Tomlinson,

Andrew Jones, Anirban Maitra, Steve Cunningham, Jacky Smith,

Catherine Fullwood, Anand Pandyan, Francis J Gilchrist

**SUPPLEMENTARY DATA**

**Figure S1: Bland-Altman comparison of predicted versus measured FRC.**

Predicted FRC was derived from previously published prediction equations applied to adult and paediatric populations separately (1, 2). Figure shows the combined datasets. There was a median difference of 0.04L (p=0.2), equivalent to 0.9% of predicted FRC.

| **Predictor** | **Dataset** | **Coefficient** | **SE** | **p-value** |
| --- | --- | --- | --- | --- |
| **Age (years)** | Whole population | 0.004 | 0.003 | 0.273 |
|  | Analysis popn.  (5-39ys) | -0.006 | 0.004 | 0.118 |
|  | Children  (5-17yrs) | -0.005 | 0.012 | 0.650 |
|  | Adults  (18+yrs) | 0.019 | 0.010 | 0.067 |
| **Height (cm)** | Whole population | -0.002 | 0.002 | 0.427 |
|  | Analysis popn. | -0.002 | 0.002 | 0.183 |
|  | Children | -0.003 | 0.002 | 0.182 |
|  | Adults | <0.001 | 0.010 | 0.961 |
| **Weight (kg)** | Whole population | <0.001 | 0.002 | 0.930 |
|  | Analysis popn. | -0.002 | 0.002 | 0.272 |
|  | Children | -0.002 | 0.002 | 0.375 |
|  | Adults | 0.009 | 0.007 | 0.242 |
| **BMI** | Whole population | 0.006 | 0.008 | 0.467 |
|  | Analysis popn. | -0.005 | 0.007 | 0.528 |
|  | Children | -0.002 | -0.010 | 0.860 |
|  | Adults | 0.023 | 0.020 | 0.246 |
| **Gender (2)** | Whole population | -0.076 | 0.077 | 0.303 |
|  | Analysis popn. | -0.113 | 0.067 | 0.095 |
|  | Children | -0.078 | 0.077 | 0.276 |
|  | Adults | -0.093 | 0.188 | 0.625 |

**Table S1: Univariate regression of factors potentially affecting lung clearance index.**

Analyses are presented for the whole population, the primary analysis population (aged 5-39ys), adults (18 yrs and over), and children (up to 17yrs). BMI: body mass index; SE: standard error.**References**

1. Goldman HI, Becklake MR. Respiratory function tests; normal values at median altitudes and the prediction of normal results. Am Rev Tuberc. 1959;79(4):457-67.

2. Polgar G, Promadhat V. Pulmonary function testing in children : techniques and standards. Philadelphia (Pa): Saunders; 1971.
