# Supplementary material for: Lung clearance index in healthy volunteers, measured using a novel portable system with a closed circuit wash-in
Source: PLoS One. 2020 Feb 25;15(2):e0229300. doi: 10.1371/journal.pone.0229300 (PMC7041809; doi:10.1371/journal.pone.0229300)
Supplement: Attachment — Submitted filename: Response to reviewers.docx [file pone.0229300.s002.docx]

**Response to reviewers: PONE-D-19-27084- Lung clearance index in healthy volunteers, measured using a novel portable system with a closed circuit wash-in**

*Editor or reviewer comments are presented in black, and response is shown in blue*

**Part 1: Editors comments**

1. Please clarify whether ethical approval was granted by University of Manchester with Lancaster Research Ethics Committee and please provide affiliation of the latter. Please also include in the Methods section participants recruitment dates.

The study was approved by an NHS research ethics committee (Lancaster REC), for approval in NHS sites. We have now made clear that this was an NHS committee. It was also reviewed by the Keele University REC as a part of the process of performing the study using Keele University staff, and in order to review the community measurements. It did not require or receive separate review by the Manchester University REC.

Recruitment period has been added.

2. Thank you for including your competing interests statement; "AH reports a collaboration agreement with Innovision ApS.
All other authors report no conflicts of interest."

Please confirm that this does not alter your adherence to all PLOS ONE policies on sharing data and materials, by including the following statement: "This does not alter our adherence to  PLOS ONE policies on sharing data and materials.” (as detailed online in our guide for authors <http://journals.plos.org/plosone/s/competing-interests>).  If there are restrictions on sharing of data and/or materials, please state these. Please note that we cannot proceed with consideration of your article until this information has been declared.

Please include your updated Competing Interests statement in your cover letter; we will change the online submission form on your behalf.

Thank you, this has been done.

3. We note that you have indicated that data from this study are available upon request. PLOS only allows data to be available upon request if there are legal or ethical restrictions on sharing data publicly. For information on unacceptable data access restrictions, please see <http://journals.plos.org/plosone/s/data-availability#loc-unacceptable-data-access-restrictions>.

At the original application we had not asked the REC whether data could be shared in this way, and have not consented volunteers to that. We do not anticipate that a REC would block this, but we would go back to them for their approval if we received a request to share data. This is standard practice where the data shared includes personal information including date of birth.

**Part 2: Response to reviewers comments**


Reviewer #1: PONE-D-19-27084
LUNG CLEARANCE INDEX IN HEALTHY VOLUNTEERS, MEASURED USING A NOVEL PORTABLE SYSTEM WITH A CLOSED CIRCUIT WASH-IN

In this manuscript Horsley et al describe MBW measurements from a substantial cohort of healthy children and adults using closed-circuit methodology to reduce overall test-time with a portable SF6 based MBW system. The authors should be congratulated for their novel work to address lengthy MBW test-time and space limitation, both of which are significant practical barriers to integration of MBW into routine clinical practice.

To my knowledge this is the first time successful use of a portable MBW device in the community has been reported. Additionally the authors report shortened test time using a closed-circuit and deep breathing to achieve rapid wash in, which appears to be feasible and repeatable within a single test occasion across the healthy cohort.

My main concerns with this study relate to the evaluation and interpretation of normative data. The authors conclude that they have established “the baseline for normative LCI” and demonstrated a stable ULN in subjects between 5-40 years, which in my opinion is not fully supported by the data as presented.

My primary concerns relate to the following:
1. ULN or Reference Data Set?
As written, the main message related to the description of this healthy data set is somewhat unclear. The title and the aims of the study suggest this is an evaluation of their healthy control group and derivation of an ULN ahead of a local longitudinal clinical study. However, a number of places throughout the manuscript, particularly in the discussion, imply that this data is generally applicable to ”provide more accurate understanding of disease severity in clinical studies conducted using this method ” (P. 10) and “establish the baseline for normative LCI”. Please clarify whether this is intended to be the first step towards developing a reference data set for this gas/device or simply to report ULN in this healthy cohort.

Thank you kindly for your detailed feedback of our manuscript.

The aim of this study was to provide normative data for LCI for researchers and clinicians using this method. There is a specific CF study associated with these data, but the use of the normal data are not limited to that study alone since there are also several other studies under way using the same method to measure LCI. The two objectives identified by the reviewer are not mutually exclusive, the data are to establish an ULN in this healthy cohort which will be used as a reference data set in future research studies.

The aims have been amended as per other comments to make this clearer.

2. MBW results are device/software specific.
In addition to being gas specific, differences in hardware, test methodology, data acquisition, software settings, calculation of outcomes, and analysis algorithms etc. all have the potential to impact LCI. I strongly disagree that the data analysis software does not impact MBW outcomes generated as is currently stated in the discussion (P.13) Previous groups have shown that MBW outcomes are significantly impacted by software (Summermatter S, et al PLoS One 2015: 10: e0132250, Anagnostopoulou P, et al Pediatr Pulmonol 2015: 50: 970-977) used to calculate results. While the consensus guidelines provide some measure of guidance there are many device specific details that are equally impactful. Strongly suggest to clearly acknowledge these limitations and clearly state that these results are are device and data analysis software version specific.

We agree that there are many different ways in which LCI can be altered, including changes in software. The point made on P13 was that of all the myriad technical and physiological variables the software should make less difference if it is designed to comply with ERS guidelines (ie two programmes doing the same thing should come up with essentially the same result). However, this is perhaps not as clear as intended, and this opinion has been removed. A more detailed reference to the provenance of the software, and the fact that the results are specific to it, has been included.

Suggest the authors consider the following:
• P6 L154: Please clearly state whether the results reported in this manuscript were derived from the Innocor device or the separate offline analysis software.

The offline software - this has been clarified

• Is the offline analysis software commercial? Please include device software version and offline analysis software version in the methods.

There is a paragraph on the software, and it has now been clarified that the software was developed “in house”. Version number and date have been added.

• Has the device, in its current form, been validated as recommended in the MBW consensus guidelines (Robinson et al, ERJ 2013)?

Yes, the device has been validated as per ERS guidelines, and the software has been designed to comply with all the ERS recommendations. This is already stated in the text (L151-2). The wash-in method was not available in 2012 and is not covered by those guidelines.

• The authors have indicated that FRC is calculated to the airway opening but have not specified calculation of LCI, ie was CEV corrected for full dead space or just re-inspired tracer gas?

This was an error in the methods description, thank you for spotting this! CEV was adjusted for equipment DS, not reinspired gas (which was used to adjust for FRC). This has now been corrected in the text.

• How do the offline results compare to the device calculations of MBW outcomes?

The comparison of the Innocor software with that used offline here is not an objective of this study. Overall the results are very comparable, as would be expected, but the use of the offline software allows more accurate correction for FGD as well as a more detailed review of the washout quality. It is not possible to easily review washouts on the Innocor’s own system and whilst it is adequate for many uses was not felt to be accurate and detailed enough for research studies using this measure as a primary outcome.

3. Is a single fixed ULN appropriate for the entire age range?
The authors aim to demonstrate that there is no linear relationship between LCI and age or height in the overall healthy cohort however, the data as presented do not clearly lead the reader to reach the same conclusions. There are some open questions that should first be addressed.
a) The authors cite previously published work as reporting a stable ULN for LCI (SF6) in healthy children (Lum et al, Eur Respir J 2013: 41: 1371-1377), a statement which could be mis-interpreted by an unfamiliar reader. Lum and colleagues report an association of LCI with height that, while minimal from approximately age 6 (~120cm), is present throughout childhood. They do suggest that a fixed ULN may be appropriate for cross-sectional analysis but caution against using a fixed upper limit for serial measurements at any age during childhood as LCI continues to change with development.
Additionally, technical and physiological factors, such as equipment specific mechanical dead space (Benseler A, Respirology 2015: 20: 459-466) and tidal volume (Yammine S, J Cyst Fibros 2014: 13: 190-197, Ratjen F, Plos One, ISSN 1932-6203, 07/2019, Volume 14, Issue 7) can dynamically influence LCI as the child grows. These effects will depend somewhat on the device and the specific population being tested and should be ruled out.

To clarify, we did not aim to show no linear relationship between LCI and age or height, we instead sought to establish what that relationship was. And the data we have presented here show no association with age or height and LCI over the ranges studied. However in the study aims we had stated that we planned to “Calculate the upper limit of normal for LCI using this device” which does perhaps imply a single value, and we have amended this to make it clearer that we had no prior preconceptions.

We are explicit that a limitation of the study is the small numbers of patients over 40 years, and can see that the few data points above this age are more disperse. Therefore we have limited our claim of a stable ULN to the age 5-40yrs. This is consistent with the data from Lum, who showed increased LCI below this, and with that from Verbanck and Htun, who reported increased LCI above about 40yrs. It is true that other researchers using other devices have shown a more linear change in LCI with age, in particular Kjellberg, and this may also be the reviewer’s experience. We have acknowledged this difference in the discussion, but we can only report what we have found. According to the data we have measured using the system described, we do not see any relationship with age or height and LCI. The reviewer is correct that changes in deadspace/tidal volume/FRC ratios with age and size will vary, but these do not appear to have been sufficiently powerful to cause changes in LCI. Had we observed such changes it would have been interesting to look at these things to investigate their influence, but in the absence of any relationship between height and LCI this is not pertinent.

Additionally we would disagree that we have been misleading about the Lum data. We have quoted the ULN from their data (relevant since this is also drawn from SF6 studies) and have referenced that LCI increases below 5 yrs, but have not explicitly claimed or inferred a fixed ULN from their data (though they did conclude this was a reasonable approach).

Suggest the authors consider the following:
• Separate evaluation of the pediatric data to determine whether there is an age/height dependency for LCI in the pediatric group. If association is found to be present, discuss the rationale for using a fixed ULN rather than prediction equations to express LCI as a z-score to account for growth in the setting of a longitudinal study. If the magnitude of the effect over the age range is small a fixed ULN may still be appropriate.

When the paediatric were data were looked at separately, there was no age or height dependency. These data have been added to the manuscript, figures are shown below.

• Is the association between LCI and age/height/BMI different between adults and children?

There is no statistically significant association at either end of the age spectrum between any of these variables. This analysis has been repeated in the entire cohort and in subgroups.

• While mean LCI was not different between adults and children, suggest to confirm that the ULN is also unchanged.

Mean LCI was unchanged (6.13 children vs 6.03 adults), but the SD in adults was slightly wider (0.53 vs 0.36). However, this is complicated by the fact that the dataset in adults is also smaller, so that the same spread will generate a slightly larger SD and hence slightly higher ULN (6.9 vs 6.7). We have now presented both options in the manuscript, but in our opinion this remains consistent with a fixed ULN to age 40yr.

• Have the authors considered the impact of equipment dead space on LCI? Particularly in children where recommended ratio of Vd to weight is no more than 2 ml/kg, total reported Vd here is 58ml making min weight 29 Kg.

At the lower end of the size range, large deadspaces will inevitably impact on gas mixing. Deadspace is always a compromise, and the volume of the system used here is little more than that of the filter and flowmeter combined. Thus in this study we have used a well-validated system with a deadspace in the smallest subjects above that deemed ideal, but which is similar to that used in previous assessments. Since we see no age or height relationship with LCI, it is reasonable to conclude that in this population the impact of deadspace on LCI is minimal.

b) In the adult population, the authors report conflicting evidence of increase in LCI with age in previously published papers with adults 17 and older (Kjellberg et al, Htun et al. and Verbanck et al.) derived from MBW using N2 as tracer gas. The authors state they have not observed a convincing increase in LCI with age in the overall adult cohort (n= 52) but acknowledge that a fixed ULN is likely inappropriate for adults over 40 (n=6) which is supported by Fig 2.
• What was the rationale for choosing age 40 as the upper cut-off in this data set?
• If the authors feel that more data is required to evaluate LCI in older adults it would be prudent to confirm that the ULN is not different when subjects >40 years are excluded.
• Given there are only 7 subjects over 40 years, and the authors suggest these data should be interpreted with caution, the first statement of the discussion may be somewhat over-stated.


40 years was chosen empirically from looking at the data as this is a fortuitously rounded point from which there are very few higher age points. Below this age there are several measurements in close proximity.

We have re-calculated the data here so that the data from those over 40yrs and over are not used in the calculation of normal ranges. These data have been retained for analyses looking at LCI dependence on age/height/BMI. This has been clearly explained in the text.

The sentence about age range has been removed

Specific comments by section:

Introduction
• P3. L48: consider additional pediatric references. More references have been added, but please note this is intended as a broad introduction to what is now a very in-depth field.
• P3. L55: define SF6 This has been done
• P3. L60-63: test time can differ with population, i.e. longer with very young children, these are generalized statements suggest to remove specific times and just point out that Spirometry is generally much faster than MBW. This has been done
• P4. L69- 72 Please briefly elaborate on how closed circuit methodology achieves faster wash-in This is covered in detail by the reference cited, but the text has been amended to make this a little clearer. For details please see Horsley AR, O'Neill K, Downey DG, Elborn JS, Bell NJ, Smith J, et al. Closed circuit rebreathing to achieve inert gas wash-in for multiple breath wash-out. ERJ Open Research. 2016;2(1).

Methods
Study Design and Recruitment
• P5L95: States “ transported in a protective case and taken to two primary schools”, previously stated primary and secondary school – please clarify. Thank you, this was an error, it was indeed one primary and one secondary school. Now corrected.
Study Assessment
Description of test procedure is unclear, suggest to edit for clarity (specific suggestions below)
• P6 L119: “Wash-in was performed from a sealed bag filled with a mixture of room air and test gas (94% O2, 1% SF6 and 5% N2O) up to a volume of 3L.” please clarify that this is total volume. This has now been done.

• P6 L123: “Initial bag volume was adjusted to be approximately equal to estimated FRC, with test gas bolus of 30-40%.” Please clarify that this refers to fraction of total bag volume. This has now been done.

• P6 L123: “These parameters could be increased if longer wash-in was required” Please clarify what parameters are referred to and why might they be adjusted?

The parameters have been clarified in the text. As stated, the reason for doing this would be to increase washin time. For example if washin O2 fell close to that of room air, or CO2 accumulated to 2% during the first test, the bag volume and/or bolus volume could be increased to ensure that this wouldn’t happen in subsequent tests.

• P6 L124: Why 5-6 deep breaths?

This is to ensure better emptying and mixing of the washin gas and improved lung ventilation with the higher concentration of tracer gas present in the first breath inspirate. This method has been previously described and validated as per the reference cited.

• P6 L125: “Once equilibrium was reached” What was absolute starting concentration?

There was not a fixed starting concentration. This would depend on the bolus fraction, bag volume and lung volume. Equilibrium was established between end washin inspired gas and end washin expired gas.

• P6 L127: “…wash-in was continued for a further 30-60 seconds.” Why?

This was to ensure confidence in washin completion. Previously reported results have shown that it is possible to reach equilibrium very quickly with this method, but that in diseased lungs washin may not be entirely complete. The method therefore requires to reach equilibrium and then continue beyond this point.

• Did all subjects use the same testing interface? Subsequently refer to different filters used-please clarify.

The same interface was used but filters differed. This has been added to the text at the end of the first paragraph on study assessment.

• P6 L137: Please describe “… detailed analysis and quality control”, what specific guidelines for quality control were used?

QC was according to recommendations in the ERS guidelines, and as described in the final paragraph of the washout analysis section.

• P6 L132: “There was no requirement for a delay between end of washout and start of next wash-in, and subjects started the next test as soon as they were able.” This is somewhat misleading - this is a consequence of using exogenous gas for MBW not specific to this device.

To clarify, we have added to this sentence “(this is a feature of all exogenous MBW systems)”

• P6 L134: “In the case of adults, visual feedback of inspiratory volumes was available to aid reproducibility of breathing patterns” What was target Vt?

Target Vt was set at their own natural Vt, since we are trying to achieve relaxed breathing, but if they were very high or low a target was set between 10-15ml/kg. This has been added to the text.

Washout Analysis
• Please clarify that offline software was used to calculate MBW outcomes reported here.

This has been done.

• P6 L151-152: The authors state that FRC is corrected for dead space and reported at the airway opening, but that CEV is adjusted to account for re-inspired gas only, please clarify that LCI is therefore reported to the gas sampling point. Please confirm that FRC used in calculation of LCI was also calculated to gsp.

This was an error in the methods description, thank you for spotting this! CEV was adjusted for equipment DS, not reinspired gas (which was used to adjust for FRC). This has now been corrected in the text.

• P6 L154: Please elaborate on quality control criteria used and rationale for using reproducibility as acceptability requirement for LCI, including references as appropriate. To my knowledge repeatability of LCI is not necessarily a criteria for disregarding data.

The reviewer is correct that there is a grey area here. There was never full consensus that differences in FRC, or what level of difference, should be included as part of QC. For this reason we have taken the more conservative value of 25% difference in FRC as being unacceptable (as per ERS guidelines and as opposed to earlier studies that used 10%). We have applied the same rule to LCI. For a study looking at healthy controls, differences in either of these repeat measures of >25% indicate that something has gone fundamentally wrong with the test, whether it can be easily identified or not. Differences of >10% triggered a closer look at the results and differences of 25% from median were therefore excluded. This may not be universally applied or may not be applied explicitly, which is why we have made this clear so that readers can understand and judge our approach.

Statistical Analysis
• How was correlation of LCI with anthropometric parameters assessed? Were any relationships between MBW outcomes and physiological factors considered? (Vt/FRC, Vd/Vt etc)

Linear regression was performed on these data, and we have now performed univariate analysis instead as per reviewer 2 recommendations. We have not looked at Vt/FRC and Vd/Vt given that we have not seen any convincing relationships with age or height and LCI. The narrow range of LCI in this study means that statistically significant relationships with other variables are unlikely to be found.

• P8 L165: Within test variability of LCI and FRC was calculated using CoV. Please provide equation. Is CoV appropriate metric when only 2 washout repeats?

Cov has been defined. This is not an ideal measurement for 2 repeats only. If there was a large proportion with only 2 measurements this would be clearly inappropriate. Excluding all these measurements however might lead to suggestion that the more variable measurements were being picked out to make the data look better. To satisfy these concerns we have now recalculated CoV for only those with 3 successful repeats, and included both sets of data in the paper.

• Did the authors assess whether there was any difference between ever/never smokers in adults?

No, the numbers were too small for this. There were only 5 ever-smokers, with very low smoking histories


FRESULTS
The result as presented are somewhat difficult to follow, suggest to edit for clarity to avoid confusion.
• Suggest clearly separating test performance (# tests/repeats attempted) from test success (# tests acceptable after QC). Possibly a flow diagram might help

The text has been made clearer and subheadings added to aid this

• P9 L185-189: Is the intention to report number of subjects who required additional (more than 3) attempts to achieve a successful test?, That is correct, and this has been made clearer.

• Please indicate the proportion of tests with 2 vs 3 successful repeats after quality control. This has been added to the text.
• Please comment on the use of repeatability of LCI/FRC as QC criteria as it relates to reporting within-test variability in the results. This point has been addressed above.
• P9 L212: Please include rationale for not evaluating relationship between LCI and BMI and FEV1/FVC in children? Please indicate data not shown or direct the reader to the results.

These data have been added


Discussion

• P11 L233: The ULN reported here is similar but lower than any previously reported in the literature using the same gas type, does the rapid technique ensure complete wash-in?
Yes, this has previously been reported on. The reference is as provided previously.

• P12 L256: Please clarify that Kjellberg et al. reported LCI derived from nitrogen based MBW

This has been done

• Please ensure that all data reported in the results are mentioned in the discussion, how do the authors interpret the FRC results? The relationship between LCI and BMI or FEV1/FVC? How should the FEV1/FVC results in adults be interpreted in context of the aging lung?

The paper is aimed at addressing the expected normal range of LCI, other methods already exist for calculation of FRC alone and it is not a measurement typically used in isolation. The discussion has been expanded to incorporate some of these additional analyses.


General Comments:
Suggest the authors revise the language to improve overall readability

The manuscript has been added to substantially, with use of subheadings in results to aid data presentation.

Figures:
• please ensure consistent nomenclature
• adjust scale so all data points are clearly visible
These have been done
Fig 3: suggest presenting different symbols for peds and adults

This has been added

Tables:
Table 1**Please ensure parameters reported in results are described in demographics**
• Please include anthropometric data (ht, wt/BMI)

Height is not useful on its own in a mixed population including adults, but we have reported BMI
• Please clearly indicate age cut-off for adults and children and check that this is consistent throughout the manuscript see P.9 L192. This has been corrected.

• Suggest to also include
o number of ever/never smokers Included
o FRC z-score We have compared FRC from MBW to that from prediction equations, this has been added to the results.
o FEV1/FRC We have included FEV1 over FVC

Reviewer #2: Horsley and colleagues present an interesting study about multiple breath washout measurements in a healthy population using a portable device based on an established MBW setup. The study is straight forward and presented in a concise way. However, there is some statistical analysis missing, which would strengthen the results.
Please find my comments below:
1. The introduction starts with a paragraph regarding LCI use, but the number of references is quite limited and not fully updated. It is important to include more references, especially recent ones, in order to catch the eye of the average reader.

More references have been added, but please note this is intended as a broad introduction to what is now a very in-depth field.

2. Introduction, line 56: Lum et al (ERJ 2013) showed also age-dependency of the LCI values, especially in infants and pre-schoolers, measuring with a mass-spectrometer using SF6.

Thank you, this reference is covered in more detail in discussion.

3. Line 60-63: Are these data based on a publication, or is it based on the authors’ personal experience? This should be clearly stated.

This was based on publications and observations, but has now been removed.

4. Line 63-66: Reference 16 refers to an inexperienced population of young CF patients. The authors state that CF children with experience in the test take 1-6 min to perform a single trial.

We have reported the study data, but made it clearer that these were test-naive patients.

5. Line 81: ‘…across a clinically-relevant age range’: what does this mean? Are the ages 5-59 clinically relevant? And, if so, why subjects below 5, or older than 59 are not relevant?

This has been removed

6. Line 123: Which parameters where used to estimate the FRC?

This was broadly estimated FRC based on patient height. This was not an accurate estimate, but in 0.5L increments a larger bag fill was used for a larger subject. For adults, in most cases, the starting bag volume was 3L. This could be adjusted up or down depending on the measured FRC of the first test.

7. Line 128: What was the maximum inspired CO2 concentration during the washin? Did it influence the breathing pattern? Did the subjects keep a tidal breathing pattern during the washin time?

CO2 was kept below 2% and monitored in real time. There were occasions when it was over this briefly but they were exceptions, and would have precipitated a change of CO2 scrubber. A detailed report of the method is covered in a separate prior publication and the method is not the subject of this paper. For details please see Horsley AR, O'Neill K, Downey DG, Elborn JS, Bell NJ, Smith J, et al. Closed circuit rebreathing to achieve inert gas wash-in for multiple breath wash-out. ERJ Open Research. 2016;2(1).

8. Did the authors perform any kind of power analysis? How did they come up with these numbers in children and adults? Was it in purpose to use less adults than children?

No power calculation was performed. The objective was to recruit as many adults and children as possible, with a target of 50 in both age groups. There was no original intent to recruit different numbers in the different age groups, but recruitment in children was enhanced by the addition of the schools arm.

9. Basic demographics (weight, height, BMI, etc) are missing from Table 1.

These have been added. We have not included height and weight as this is a mixed adults and paeds population, making these data hard to interpret, but we have included BMI.

10. Table 1: Which reference values were used for FEV1? The range is very high, and some FEV1 values seem to be very low. How do the authors comment on that, especially in a healthy cohort?

We have used the GLI equations, and have now added this to the text (the omission was an oversight). We included the full range in the original table 1, which was perhaps misleading. Healthy subjects are capable of obtaining outlier values like this, but it was not the norm and median z score was -0.24. We have quoted IQR in the current table.

11. How was the CoV calculated? This has been covered above and is now detailed in the methods.

12. In order to investigate the influence of demographics or other factors on the LCI, several studies have used more sophisticated statistical methods, like regression models. What are the effects of age, height, sex, etc on the LCI, using a regression analysis? What other factors could potentially influence this outcome?

We have amended the analysis to use univariate regression analysis of relevant demographic factors, and have not found any significant (or near-significant) associations with any of these. In the absence of these associations we have not conducted multiple regression analysis. There were only small numbers of subjects from non-Caucasian backgrounds or ex-smokers, and these have not been included as separate factors.

13. The overall time of the test duration, although interesting, does not give exact information about the expected duration of a single trial, especially in children, where intervals between trials can be larger. Is there any way to estimate the duration of a single trial?

No, we are unable to separate out these data. The data shown are for the total file length though, including any additional repeat tests required, and therefore represent a real-world estimation of test time.

14. Although LCI is the primary outcome of the study, FRC calculation is critical for the estimation of the LCI. Apart from figure 4, there is no analysis showing effects of demographics and/or other factors on FRC. How do the authors discuss their findings in FRC in comparison to previous studies? Is the FRC critical for the duration of the test?

Since this study aims to focus on LCI, the derivation of new equations for lung volumes are out of scope. The reason for showing the FRC data were to make clear that the lung volumes were increasing as expected and within expected physiological ranges.

However, the reviewer makes a very valid point, and since errors in FRC with other MBW systems are only now being recognised it is important to demonstrate that the FRC are not deviating excessively from expected values. We have therefore included a comparison of our FRC values with those derived from prediction equations. FRC repeats are often highly variable between testing sessions, even with MBW, and the spread of data is therefore relatively wide. However, we show no consistent deviation from a zero difference, with a bias of only 0.06 L or 0.9% of predicted FRC. We have added these data to the results and the discussion. The graph is provided below but we do not propose to include this in the manuscript. This shows a Bland-Altman comparison between predicted and measured FRCs, across the whole dataset. Dotted lines represent mean bias and limits of agreement.

15. It would worth to mention and compare the results with other studies reporting reference values for LCI, either with the same (e.g. Fuchs S and colleagues, Pediatric Pulmonology 2009) or with other tracer gases.

This has already been covered extensively in the discussion, with a focus on comparable technologies and recent studies. The reference provided has also been added.
